# Noise Annoyance Is Associated with Depression and Anxiety in the General Population- The Contribution of Aircraft Noise

**DOI:** 10.1371/journal.pone.0155357

**Published:** 2016-05-19

**Authors:** Manfred E. Beutel, Claus Jünger, Eva M. Klein, Philipp Wild, Karl Lackner, Maria Blettner, Harald Binder, Matthias Michal, Jörg Wiltink, Elmar Brähler, Thomas Münzel

**Affiliations:** 1 Department of Psychosomatic Medicine and Psychotherapy, University Medical Center of the Johannes Gutenberg University Mainz, Mainz, Germany; 2 Medical Clinic for Cardiology, Angiology and Intensive Care Medicine, University Medical Center of the Johannes Gutenberg University Mainz, Mainz, Germany; 3 Preventive Cardiology and Preventive Medicine, Department of Medicine 2, University Medical Center of the Johannes Gutenberg University Mainz, Mainz, Germany; 4 Center for Thrombosis and Hemostasis, University Medical Center of the Johannes Gutenberg University Mainz, Mainz, Germany; 5 German Center for Cardiovascular Research (DZHK), partner site Rhine Main, University Medical Center of the Johannes Gutenberg University Mainz, Mainz, Germany; 6 Institute for Clinical Chemistry and Laboratory Medicine, Germany, University Medical Center of the Johannes Gutenberg University Mainz, Mainz, Germany; 7 Institute for Medical Biostatistics, Epidemiology and Informatics (IMBEI), University Medical Center of the Johannes Gutenberg University Mainz, Mainz, Germany; Johannes-Gutenberg University of Mainz, GERMANY

## Abstract

**Background:**

While noise annoyance has become recognized as an important environmental stressor, its association to mental health has hardly been studied. We therefore determined the association of noise annoyance to anxiety and depression and explored the contribution of diverse environmental sources to overall noise annoyance.

**Patients and Methods:**

We investigated cross-sectional data of n = 15.010 participants of the Gutenberg Health Study (GHS), a population-based, prospective, single-center cohort study in Mid-Germany (age 35 to 74 years). Noise annoyance was assessed separately for road traffic, aircraft, railways, industrial, neighborhood indoor and outdoor noise (“during the day”; “in your sleep”) on 5-point scales (“not at all” to “extremely”); depression and anxiety were assessed by the PHQ-9, resp. GAD-2.

**Results:**

Depression and anxiety increased with the degree of overall noise annoyance. Compared to no annoyance, prevalence ratios for depression, respectively anxiety increased from moderate (PR depression 1.20; 95%CI 1.00 to 1.45; PR anxiety 1.42; 95% CI 1.15 to 1.74) to extreme annoyance (PR depression 1.97; 95%CI 1.62 to 2.39; PR anxiety 2.14; 95% CI 1.71 to 2.67). Compared to other sources, aircraft noise annoyance was prominent affecting almost 60% of the population.

**Interpretation:**

Strong noise annoyance was associated with a two-fold higher prevalence of depression and anxiety in the general population. While we could not relate annoyance due to aircraft noise directly to depression and anxiety, we established that it was the major source of annoyance in the sample, exceeding the other sources in those strongly annoyed. Prospective follow-up data will address the issue of causal relationships between annoyance and mental health.

## Introduction

Noise, defined as ‘unwanted sound’ has gradually become increasingly acknowledged as an environmental stressor and as a nuisance [1]. Non-auditory effects of noise occur at levels far below those required to damage the hearing organ. Although people tend to habituate to noise exposure, the degree of habituation differs substantially between individuals and is rarely complete. If exposure to noise is chronic and exceeds certain levels, adverse health outcomes can be seen. Meanwhile numerous studies have shown that noise contributes to sleep disturbance, to the development of arterial hypertension, ischemic heart disease, heart failure, arrhythmia, metabolic syndrome and stroke (for review see [2]) and also to learning, respectively emotional difficulties in children [3].

According to the noise reaction model, two principal pathways are relevant for the development of adverse health effects of noise [4]. These refer to the ‘direct’ and the ‘indirect’ arousal and activation of the organism. The ‘direct’ pathway is determined by the instantaneous interaction of the acoustic nerve with different structures of the central nervous system. The ‘indirect’ pathway refers to the cognitive perception of the sound, its cortical activation and related emotional responses such as annoyance. Both, noise level and noise annoyance have been shown to be associated with cardiovascular disorders. Both reaction chains may initiate physiological stress reactions. The activation of fight–flight and defeat reactions is thought to involve subcortical regions of the brain like the hypothalamus, which has inputs to the autonomic nervous system, the endocrine, and the limbic system. If experienced chronically, stress caused by annoyance may even trigger the development of cardiovascular disease [2].

Annoyance is the most prevalent community response in a population exposed to environmental noise. Noise annoyance can result from interference with daily activities, feelings, thoughts, sleep, or rest, and may be accompanied by negative emotional responses, such as irritability, distress, exhaustion, a wish to escape the noise and other stress-related symptoms [5, 6]. Severe annoyance has been associated with reduced well-being and health, and because of the high number of people affected, annoyance contributes substantially to the burden of disease from environmental noise. It is estimated that DALYs (disability adjusted life years) from environmental noise in the Western European countries are 61,000 years for ischemic heart disease, 45,000 years for cognitive impairment for children, 903,000 for sleep disturbance, 22,000 years for tinnitus and 587,000 for annoyance solely http://www.euro.who.int/__data/assets/pdf_file/0008/136466/e94888.pdf.

While there is a clear relationship between the objective measurement of noise and annoyance, individual factors such as noise sensitivity, internal states (genetic, physiological, psychological, life style) that increase individuals’ reactivity to noise in general [7] may play an important role [8].

The burden of mental disorders is quite high and disabling. Depression and anxiety disorders rank among the disorders with the strongest impact, as reflected by years lived with disability and reduced quality of life [9]. Comorbid somatic and mental disorders carry higher burdens when compared to somatic disorders without mental comorbidities and mental disorders without somatic comorbidities [10]. Central to most contemporary etiological theories is the notion that stress can initiate cognitive and biological processes that increase the risk for depression and for anxiety disorders [11]. Thus, it is surprising that the effect of noise on mental health has so far been infrequently studied in adults.

In a cross-sectional survey of residential areas stratified by aircraft noise level, [6, 12], annoyance was associated with an increase of noise level and with acute mental and physical symptoms. However, chronic symptoms were higher in the areas with comparatively lower noise level. Regardless of the level of noise, persons with high annoyance reported more mental and physical symptoms and used more psychotropic drugs, general practice and outpatient services [13]. In their review, van Kamp and Davies [7] concluded that individuals with mental disorders constitute a risk group for heightened noise sensitivity (along with chronically somatic ill, people suffering from tinnitus, shift workers, fetuses and neonates), which increases their risk for adverse health effects of noise. Preliminary studies indicated different effects of specific sources of traffic noise on attitudes and annoyance responses [14, 15]. In general, aircraft noise has been found more annoying, and with stronger effects on sleep, than road and railway noise [16]. In a large a population-based, prospective, observational single-center cohort study in the Rhine-Main-Region in western Mid-Germany we recently studied a sample of 15,010 participants drawn randomly from the local registry in the city of Mainz and the district of Mainz-Bingen [17]. Some of these areas are affected by a direct neighborhood to the Frankfurt Airport flight pattern; others are expected to be affected more by road traffic (e.g. motorways) or railway noise. With the present studies we were specifically interested in the relationship between noise annoyance and mental health by assessing total noise annoyance during day and night. In a second step we wanted to differentiate between the sources of extreme annoyance according to a broad range of sources (road, aircraft, train, industrial, neighborhood, etc.).

The specific questions we wanted to address with the current studies were:

1. Is noise annoyance in general associated with anxiety and depression?
2. How annoying are different sources of noise?

## Methods

### Study sample

We investigated cross-sectional data of n = 15.010 participants enrolled in the Gutenberg Health Study (GHS) from April 2007 to April 2012 [17]. The GHS is a population-based, prospective, observational single-center cohort study in the Rhein-Main-Region in western Mid-Germany. The study and its procedure have been approved by the ethics committee of the Statutory Physician Board of the State of Rhineland-Palatinate and by the local and federal data safety commissioners. Participation was voluntary and written informed consent was obtained from each subject upon entry into the study. The primary aim of the study was to evaluate and improve cardiovascular risk stratification. The sample was drawn randomly from the local registry in the city of Mainz and the district of Mainz-Bingen. The sample was stratified 1:1 for gender and residence and in equal strata for decades of age. Inclusion criteria were age 35 to 74 years and written informed consent. Persons with insufficient knowledge of German language, or physical and mental inability to participate were excluded. Based on the interim analysis 5.8% were excluded because of the exclusion criteria. The response rate (defined as the recruitment efficacy proportion, i.e. the number of persons with participation in or appointment for the baseline examination divided by the sum of number of persons with participation in or appointment for the baseline examination plus those with refusal and those who were not contactable) was 60.3%. A total of 14.635 participants answered the noise annoyance items. Mean age was 54.9 (±11.1); 49.4% were female.

### Materials and assessment

The 5-hour baseline-examination in the study center comprised evaluation of prevalent classical cardiovascular risk factors and clinical variables, a computer-assisted personal interview, laboratory examinations from a venous blood sample, blood pressure and anthropometric measurements. In general, all examinations were performed according to standard operating procedures by certified medical technical assistants

### Questionnaires

Noise annoyance was assessed in analogy to Felscher-Suhr et. al. [18] by single questions in the format: “How annoyed have you been in the past years by …”? Six potential sources of noise annoyance (road traffic, aircraft, railways, industrial/construction, neighborhood indoor and outdoor) were separately rated “during the day” and “in your sleep”. Ratings were done on a five-point scale (“not, slightly, moderately, strongly, extremely”).

Depression was measured by the Patient Health Questionnaire (PHQ-9), which quantifies the frequency of being bothered by each of the 9 diagnostic criteria of Major Depression over the past 2 weeks. Responses are summed to create a score between 0 and 27 points. A PHQ-9 sum score of ≥ 10 was used for the definition of caseness for depression yielding a sensitivity of 81% and a specificity of 82% for any depressive disorder[19].

Generalized anxiety was assessed with the two screening items of the short form of the GAD-7 (Generalized Anxiety Disorder [GAD]-7 Scale) [20]. On the GAD, subjects rated “Feeling nervous, anxious or on edge” and “Not being able to stop or control worrying” by 0 = “not at all”, 1 = “several days”, 2 = “over half the days”, and 3 = “nearly every day”. A sum score of 3 and more (range 0–6) out of these two items indicates generalized anxiety with good sensitivity (86%) and specificity (83%). Both the GAD-7 and GAD-2 have been shown to perform well as screening tools for all anxiety disorders [20].

### Computer-assisted personal Interview

During the computer-assisted personal interview participants were asked whether they had ever received the definite diagnosis of any depressive disorder (medical history of lifetime diagnosis of any depressive disorder, MH of Depression) by a physician. The socioeconomic status (SES) was defined according to Lampert’s and Kroll’s scores with a range from 3 to 27 (3 indicates the lowest SES and 27 the highest SES) [21].

### Statistical analysis

Variables were reported as absolute numbers, percentages or means with standard deviations or medians with 25^th^ and 75^th^ percentiles as appropriate. As we were interested in total noise annoyance, we used the highest annoyance rating of all categories of noise (aircraft, road traffic, etc.) as an indicator of overall noise annoyance, regardless of whether it affected daytime or sleep. Comparisons between groups were done with Cochran-Armitage test for trend for categorical variables and with Jonckheere-Terpstra test for continuous variables as appropriate. To investigate the association between overall annoyance and depression (PHQ-9 ≥10), respectively generalized anxiety (GAD-2 ≥3) multiple generalized linear models with a binominal distribution and a log link function adjusted for sex, age and socioeconomic status were used. All reported *p*-values corresponded to 2-tailed tests. As this is an explorative study no adjustments for multiple testing have been done. *P*-values were given for descriptive reasons only. Due to the large number of tests, *p*-values should be interpreted with caution and in connection with effect estimates. Statistical analyses were performed using SAS for Windows 9.4 TS Level 1M1 (SAS Institute Inc.) Cary, NC, USA.

## Results

Table 1 shows depression and anxiety according to degree of noise annoyance. Of the study participants, 20.7% reported no, 26.6% slight, 25.0% moderate, 17.3% strong, 10.5% extreme annoyance by noise. Mean depression and anxiety scores increased steadily from 3.5 to 5.1, respectively 0.7 to 1.1, with the degree of annoyance. The rates of clinically significant anxiety (GAD-2≥3) and depression (PHQ-9≥10) and medical diagnoses of depression, respectively anxiety, also increased steadily.

**Table 1:** Depression and anxiety according to the extent of total noise annoyance.

	*No*		*Slight*		*Moderate*		*Strong*		*Extreme*		*p*-value
	n *= 3024 (20*.*7%)*		n *= 3895 (26*.*6%)*		n *= 3654 (25*.*0%)*		n *= 2536 (17*.*3%)*		n *= 1530 (10*.*5%)*		
	*M*	*CI95%*	*M*	*CI95%*	*M*	*CI95%*	*M*	*CI95%*	*M*	*CI95%*	
Depression Score (PHQ- 9)	3.5	[3.4; 3.6]	3.7	[3.6; 3.8]	4.1	[4.0;4.2]	4.6	[4.4;4.7]	5.0	[4.8;5.2]	<.0001^*1)*^
Anxiety Score (GAD-2)	0.7	[0.7; 0.7]	0.8	[.8; .8]	0.9	[0.9; 0.9]	1.0	[0.9; 1.0]	1.1	[1.0;1 .2]	<.0001^*1)*^
	%		%		%		%		%		
Depression (PHQ- 9 ≥10)	6.1		5.8		7.2		9.6		12.0		<.0001^*2)*^
Anxiety (GAD-2 ≥3)	4.5		5.4		6.5		8.0		10.0		<.0001^*2)*^
Depression (medical diagnosis)	10.1		10.7		11.2		14.2		14.8		<.0001^*2)*^
Anxiety (medical diagnosis)	6.3		6.0		7.2		8.3		9.9		<.0001^*2)*^

**Fig 1:**
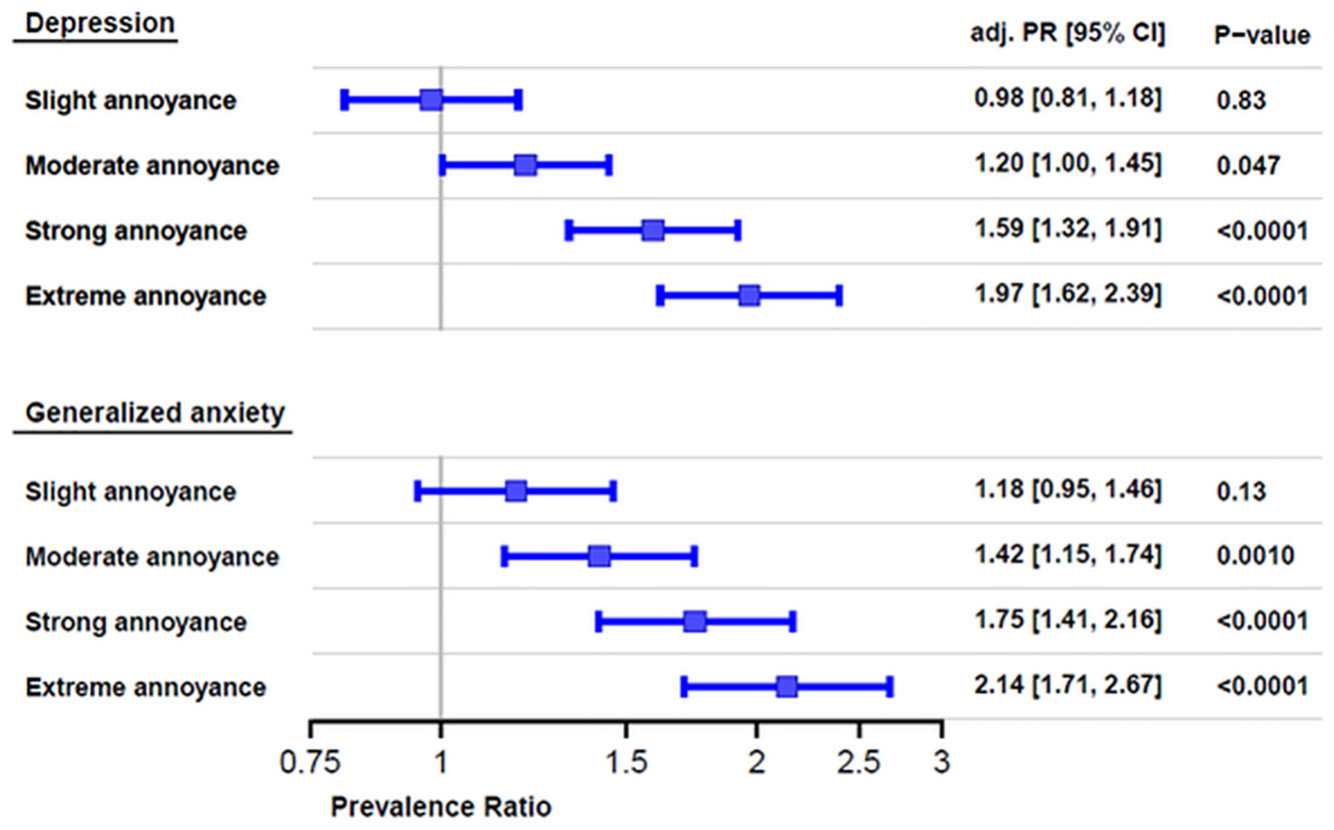
Association between noise annoyance, depression and anxiety. Note. Multiple generalized linear models with a binominal distribution and a log link function adjusted for sex, age and socioeconomic status were used.

In order to determine the associations between noise annoyance, depression and anxiety, we performed a logistic regression controlling for sex, age and socioeconomic status (Fig 1). Compared to no annoyance, the odds ratio for depression increased steadily starting from moderate (1.22; 95%CI 1.00 to 1.49) to extreme annoyance, which had a 2.12 fold (95%CI 1.71 to 2.64) likelihood of depression. Correspondingly, the likelihood of anxiety increased from moderate (1.45fold; 95% CI 1.16 to 1.81) to extreme annoyance (2.28 fold; 95% CI 1.79 to 2.91).

**Fig 2:**
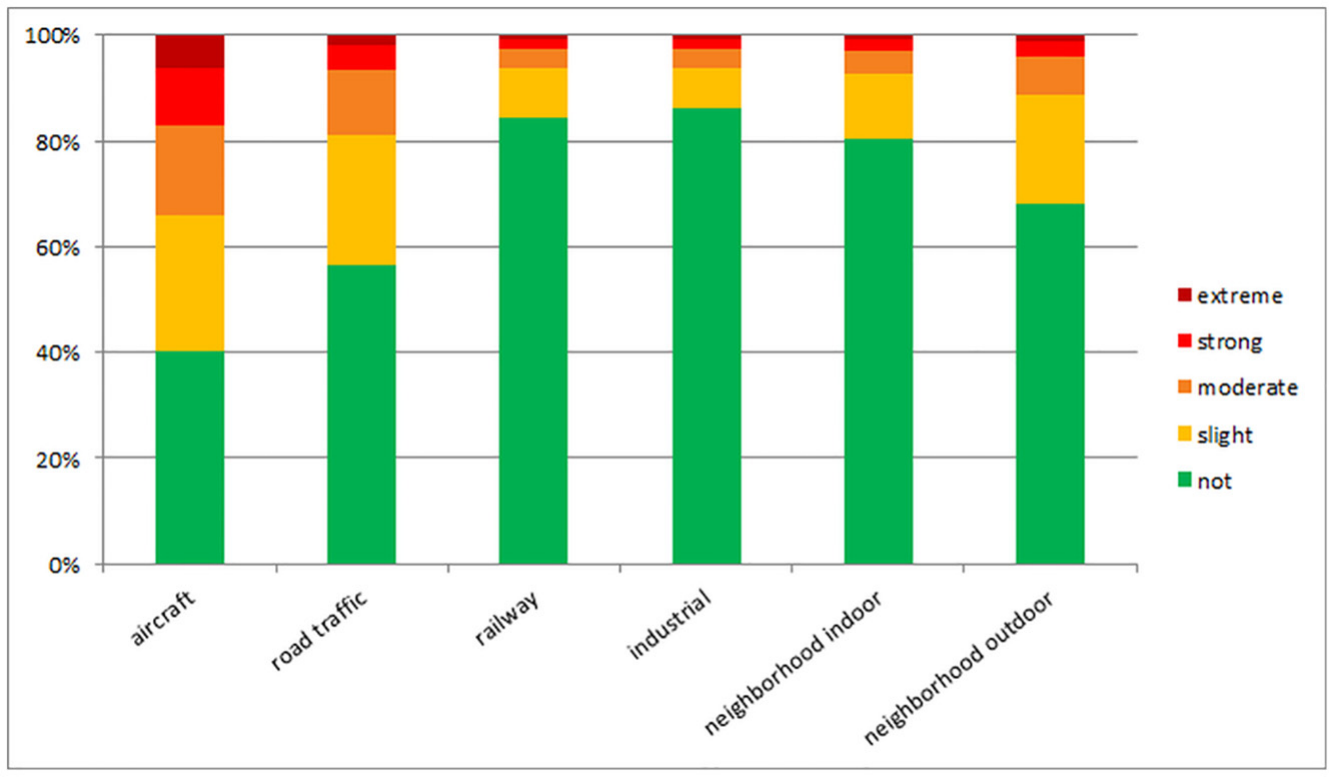
Degrees of overall annoyance according to different sources of noise.

As Fig 2 shows, the degree of annoyance was highest due to aircraft noise (affecting 59.9% of the population to some degree and 6.4% extremely), followed by road traffic (43.5%; 1.9%), neighborhood outdoor (31.8%/ 1.2%), indoor (19.6%; 0.9%), railway (15.8%/0.7%) and industrial noise (19.6%/ 0.9%).

**Fig 3:**
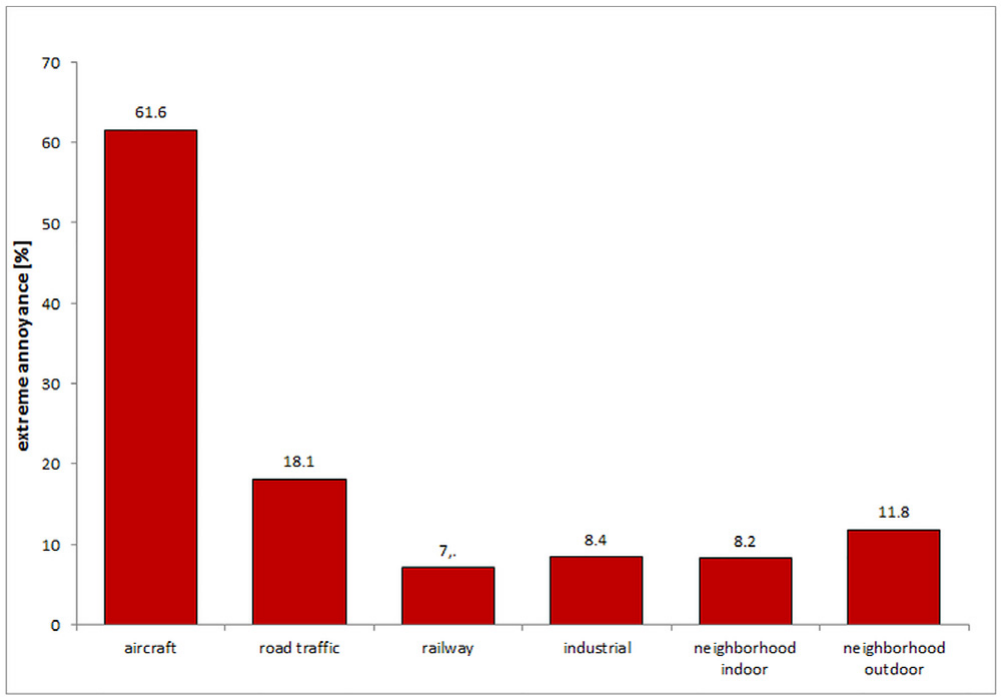
Sources of extreme annoyance (*N* = 1530).

Fig 3 summarizes the sources of extreme noise annoyance. Clearly, aircraft noise has turned out the leading source with over 60%, followed by road traffic (18.1%), neighborhood (outdoor), industrial, neighborhood (indoor) and railway noise.

## Discussion

The present study shows that the degree of noise annoyance reported by people living in the vicinity of the Frankfurt Airport and taking part in the Gutenberg Health Study (GHS) is strongly associated with the degree of depression and anxiety. Compared to other sources, annoyance by aircraft noise was prominent affecting almost 60% of the population and also accounted mostly for extreme degrees of annoyance. The vast majority of the large population-based sample reported noise annoyance; only 20.7% reported no annoyance. More than half (52.8%) of the population were at least moderately annoyed. Importantly, by adjusting for age, sex and socioeconomic status we could rule out possible demographic effects or effects of noisy low-socioeconomic environments.

When we looked at the sources of annoyance, the majority of participants reported annoyance due to aircraft, which exceeded all other sources of noise. Aircraft noise has also turned out as the leading source of extreme annoyance (with over 60%), followed by road traffic, neighborhood (outdoor), industrial, neighborhood (indoor) and railway noise. With respect to aircraft but not road traffic noise, there has been a substantial increase in annoyance reactions over the years at the same intensity of noise [5]. Babisch et al. recently demonstrated that annoyance ratings in the HYENA study were clearly higher than predicted by the EU standard curves [5].

Anxiety and depression belong to the most frequent and impairing conditions in the community; the 12-month prevalence of anxiety disorders was 14.0% and major depressive disorders afflicted 6.9% in Europe according to large epidemiological studies. Nearly one in four women and one in six men experience depression during their lifetime. Nearly twice the number of women reported anxiety disorders or depression compared to men. Living alone, a lower educational level, and a low socioeconomic status have been identified as risk factors for anxiety disorders and depression [10].

Occupational noise and depression have been studied most intensely, e.g. a large recent Korean survey by Yoon et al. [22] found that occupational noise annoyance was strongly associated to depressive symptoms and suicidal ideation in men and women (odds ratios OR 1.41 to 1.76). An Egyptian study of airport workers found hearing impairment, raised blood pressure, headaches, disturbed sleep, and symptoms of anxiety were more prominent among the noise exposed workers than the controls [23].

Findings regarding traffic noise, depression and anxiety are rather limited and somewhat controversial. In one study the association between traffic noise and depression was due to the link between noisy, low-socio-demographic environments and mental health [24]; other studies [6, 12, 13] found that the association between noise and mental symptoms was mediated by annoyance, respectively noise sensitivity [7]. A large, recent Danish study by Roswall et al. found that calculated residential exposure to road traffic and railway noise had significant, but small negative effects on quality of life [25].

Regarding aircraft noise, indirect evidence for a potential negative effect on mental health, respectively sleep was provided by Floud et al. [26] Aircraft noise during the day (OR = 1.28) and the night (OR = 1.27) was significantly associated with the intake of anxiolytics; the effects for annoyance were considerably stronger (OR = 1.78; OR = 1.77) [26]. The intake of antidepressants was only increased by noise annoyance at night (OR = 1.59) [26]. Based on health insurance data, the large case control study [27] found an increased likelihood of inpatient treatment for depression in women (neither for men nor anxiety disorders) exposed to intensive night time aircraft noise; the same applied to the intake of antidepressants. A small case control study by Hardoy et al. [28] showed an increased risk for long-lasting syndromal anxiety states (Generalized Anxiety Disorder and Anxiety Disorder NOS) in participants exposed to aircraft noise.

As in the international literature, substantial proportions of the population were afflicted with depression (6.6%) and anxiety disorders (7.3%). Previous medical diagnoses of depression were reported by a total of 12.0%; medical diagnoses of anxiety disorder by 7.3%. According to our findings, the intensity of depression and anxiety, and also the proportions of medical diagnoses of depression, respectively anxiety increased with noise annoyance.

Importantly, the magnitude of the association between noise and depression/anxiety was comparable to the previously reported association of depression with coronary heart disease [2, 29–31]. Thus, in addition to direct negative cardiovascular effects of noise [2, 29–31] it is tempting to speculate there might be some indirect adverse effect of noise in inducing cardiovascular disease via causing depression and anxiety disorders.

More than half (52.8%) of the population were at least moderately annoyed and thus at an increased risk for depression and for generalized anxiety; the intensity of the risk increased steadily with the degree of annoyance amounting to a more than two-fold risk (2.12 for depression, 2.28 for generalized anxiety) at the level of extreme annoyance.

The demonstration of an association of noise annoyance with current depression and anxiety disorder is compatible with the hypothesis that annoyance induces stress, which in turn may precipitate or even worsen already existing depression and anxiety disorders. However, given the cross-sectional nature of our study and previous findings [6, 12, 13] we cannot preclude that depression, respectively anxiety disorders may also indicate a heightened noise sensitivity. Thus, existing mental disease may deteriorate due to noise [7]. As anxiety and depression are among the most frequent and burdening diseases in the general population, substantial parts of the population may thus be particularly vulnerable to environmental noise. Alternatively, increased annoyance may be a symptom of depression and anxiety related to the increased irritability sometimes found in these conditions or to the negativity to one's surroundings found in depression.

Strengths of our study are the large sample size of over 15.000 residents and the representative nature of the sample. While previous surveys usually assessed one or two kinds of noise (e.g. road, air traffic or occupational), we were able to cover a broad range of sources including neighborhood noise indoors and outdoors, and we were able to adjust for socio economic status. Limitations of the study refer to the age range (35 to 74 years) and the participation rate. Noise annoyance may be lower in younger people [7]. Further analyses will help to specify the contributions of specific sources of noise to annoyance during the day and in the sleep. The focus of this paper was on subjective annoyance, and we could not relate it to objective measures of noise exposition. Based on the cross-sectional nature of our data we cannot make causal statements on the relationship of annoyance to mental health, i.e. participants with mental health problems may be more noise sensitive and report higher annoyance [7]. We expect further clarification of these issues from our regular follow-up assessments of the 15,000 included cohort subjects in order to address the issue of causal relationships between annoyance and mental health.
